# Supplementary material for: Majority of human circulating IgG plasmablasts stop blasting in a cell-free pro-survival culture
Source: Sci Rep. 2024 Feb 13;14:3616. doi: 10.1038/s41598-024-53977-2 (PMC10864258; doi:10.1038/s41598-024-53977-2)
Supplement: Supplementary file 1 — Supplementary Figures. [file 41598_2024_53977_MOESM1_ESM.pdf]

Supplementary figures (S1-S6)

Majority of human circulating IgG plasmablasts stop blasting in a cell-free pro-survival culture

Doan C. Nguyen[1], Celia Saney[1#], Ian T. Hentenaar[1], Monica Cabrera-Mora[1], Violeta Capric[1], Matthew C. Woodruff[2,3], Joel Andrews[4], Sagar Lonial[4], Ignacio Sanz[2,3], F. Eun-Hyung Lee[1,3*]

Suppl. Fig. S1. BrdU flow cytometric analysis of cultured blood ASC and bulk IgG ELISpots. (**a**) Cultured blood ASC were assayed for IgG secretion by ELISpots. (**b**) Quantitation of IgG secretory ASC in (**a**). (**c**) General gating strategy used for flow cytometric analysis to assess cell cycle phase in cultured blood ASC. The end panel represents a diagram for phase-flow BrdU cell proliferation with the individual cell-cycle phases (S, BrdU+; G0/G1, 2N and BrdU-; and G2/M, 4N and BrdU-) indicated. (**d**) Flow cytometric analysis of BrdU incorporation in blood ASC at day 22 in culture. The percentage of ASC in S-phase was estimated by the amount of BrdU detected (analyzed with apoptotic cells excluded). (**e**) Blood ASC at day 22 in culture were assayed for IgG secretion by ELISpots. (**f**) Quantitation of IgG secretory ASC in (**e**). In (**a**,**e**): Representative IgG ELISpot images shown. The number below indicates the calculated input number of ASC that were measured at day 0.

Suppl. Fig. S2. An illustration for the OptoSelect®1500 chip and schematic diagrams of its interior portions. Adapted with permission from Bruker Cellular Analysis, Inc. (formally Berkeley Lights, Inc.).

Suppl. Fig. S3. Timing of the few IgG secreting blood ASC that divide. (**a**) Majority of blood ASC do not divide when observed to day 15. Image series displaying a representative single blood ASC with ongoing Ig secretion and no evidence of division ((bright field) as far as day 15. (**b**,**c**) Representative blood ASC undergoing division within 8h (**b**) or 36h (**c**). Cell division was assessed by visualization of cell addition ((bright field) over the course of the culture. In (**b**,**c**): Ref: reference image show single cell penned at time of loading.

Suppl. Fig. S4. BrdU flow cytometric analysis of cultured BM LLPC and bulk IgG ELISpot assays. (**a**) Ki-67 flow cytometry assay controls using BMMC were stained with isotype controls or anti-Ki-67 antibody. The numbers indicate the percentages of the cells positive for Ki-67. (**b,c**) IgG ELISpots of cultured BM LLPC. Quantitation of IgG secretory BM LLPC in (**b**). (**d**) Gating strategy used for flow cytometric analysis to assess cell cycle phases in cultured BM LLPC. The last panel represents a diagram for phase-flow BrdU cell proliferation with the individual cell-cycle phases (S, BrdU+; G0/G1, 2N and BrdU-; and G2/M, 4N and BrdU-) indicated. (**e**) Flow cytometric analysis of Ki-67 expression in BM LLPC at d56 in culture (with unstimulated naïve B cells at d1 as a non-proliferative control). The numbers indicate the percentages of the cells positive for Ki-67. (**f**) Flow cytometric analysis of BrdU incorporation in BM LLPC at d56. The percentage of ASC in S-phase was estimated by the amount of BrdU detected (analyzed with apoptotic cells excluded). (**g, h**) BM LLPC at d56 were assayed for IgG secretion by ELISpots. Quantitation of IgG secretory BM LLPC in (**g**). In (**b**,**g**): Representative IgG ELISpot images shown. The number below indicates the calculated input number of ASC at day 0.

Suppl. Fig. S5. Mature BM ASC with Ig secretion do not divide even up to day 14 in single cell cultures. (**a**,**b**) Image series displaying a single BM PopD (LLPC) (**a**) or BM PopB (**b**); 2 nearby pens each with a single ASC) with ongoing Ig secretion showing no evidence of division ((bright field) by day 4. (**c**) Quantitation (left y axis) and frequency (right y axis) of dividing and non-dividing single cell BM PopB across 4 independent experiments. Div, dividing; Non-div, no dividing. In (**a**,**b**): Cell division was assessed by visualization of cell addition ((bright field) over the course of the culture. In (**c**): Div, dividing; Non-div, no dividing.

Suppl. Fig. S6. Limited sensitivity by the optofluidic platform (the Lightning machine) in capturing single ASC IgG secretion (performed over 80 minutes). (**a**) Use of bulk ELISpots to detect total IgG secretion from (left) an established human IgG-producing myeloma cell line, namely ARH-77 (1,000 cells, cultured in RPMI 1640 supplemented with 10% FBS; see Methods), and (right) primary, healthy blood ASC isolated from Tdap-vaccinated adults (800 cells, cultured in MSC secretome). All ELISpots were performed with cells incubated for 18-24h. (**b**) Use of the Lightning (under the 4x objective) to capture total IgG secretion from (left) ARH-77 and (right) healthy blood ASC isolated from Tdap-vaccinated adults (as done with the ELISpots). Lower panel, bright field; upper panel, IgG capture. All Lightning capture assays were performed within a time window of 80 minutes. The primary, healthy blood ASC assays served as a positive control.

Supplementary figures (S1-S6)

Suppl. Fig. S1.

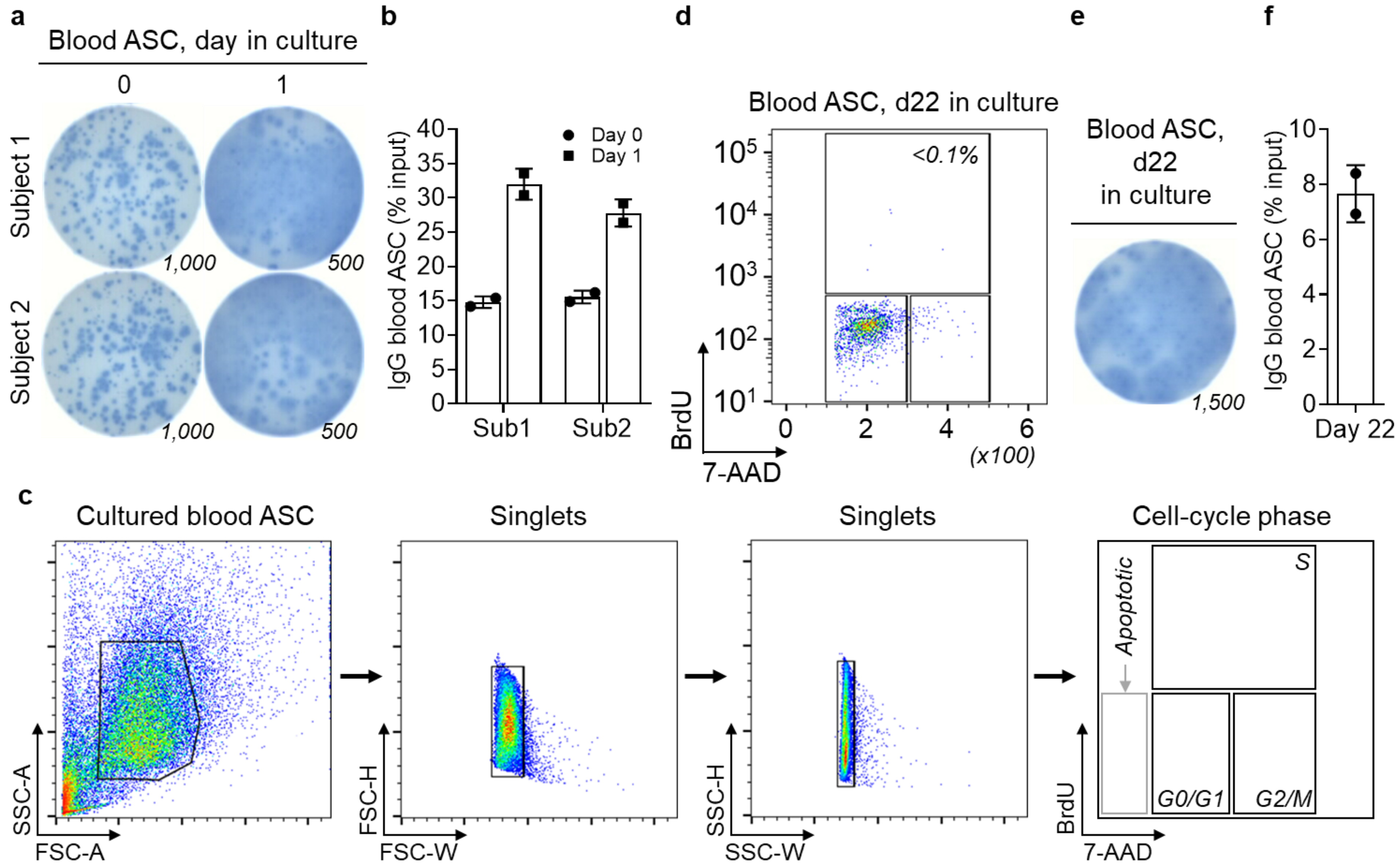

Suppl. Fig. S2.

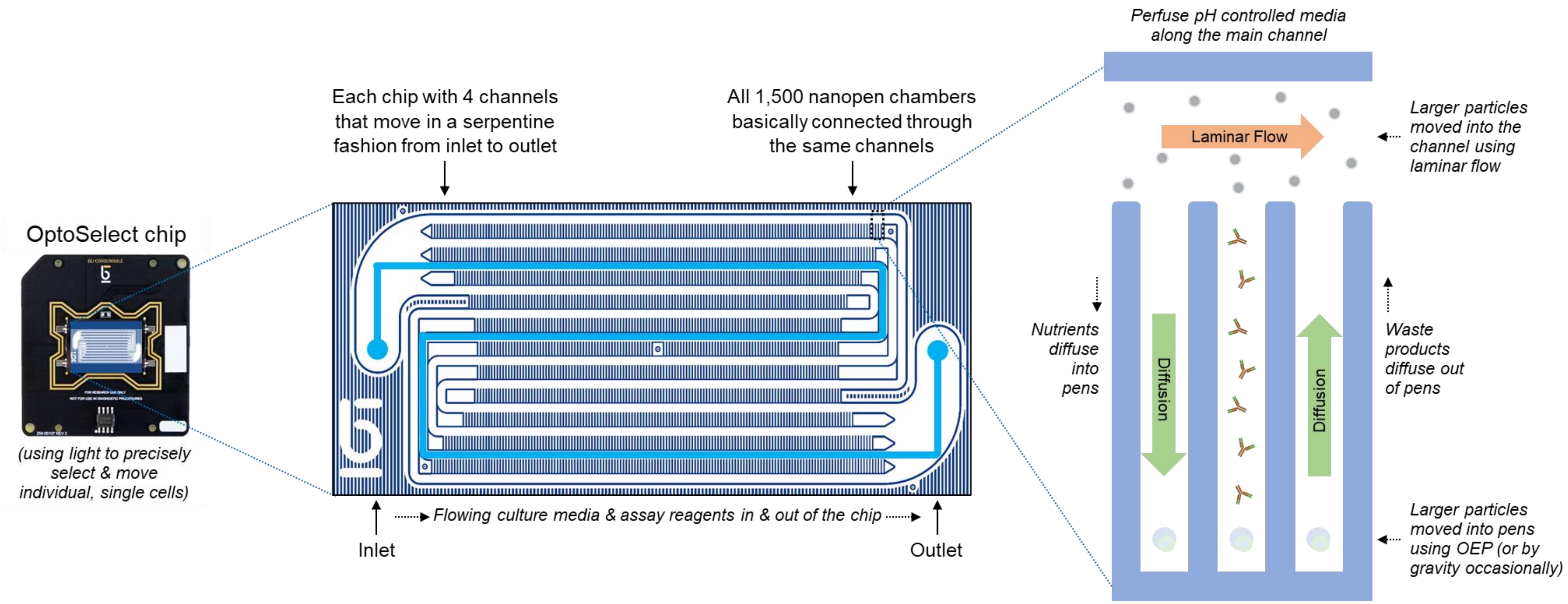
OptoSelect chip
(using light to precisely select & move individual, single cells)
Each chip with 4 channels that move in a serpentine fashion from inlet to outlet
All 1,500 nanopen chambers basically connected through the same channels
Inlet
Flowing culture media & assay reagents in & out of the chip
Outlet
Perfuse pH controlled media along the main channel
Laminar Flow
Larger particles moved into the channel using laminar flow
Nutrients diffuse into pens
Diffusion
Diffusion
Waste products diffuse out of pens
Larger particles moved into pens using OEP (or by gravity occasionally)

Suppl. Fig. S3.

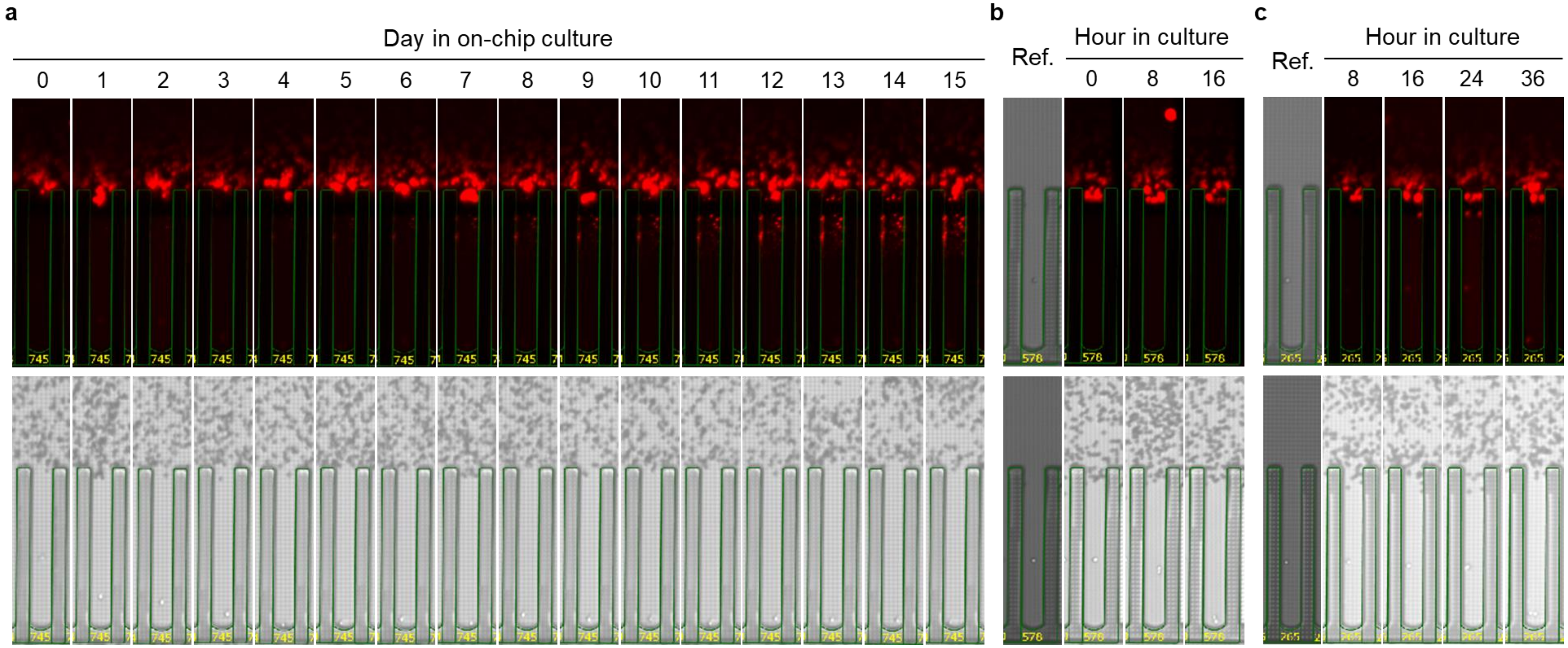
a
Day in on-chip culture
0 1 2 3 4 5 6 7 8 9 10 11 12 13 14 15
745
b
Ref.
Hour in culture
0 8 16
578
c
Ref.
Hour in culture
8 16 24 36
265

Suppl. Fig. S4.

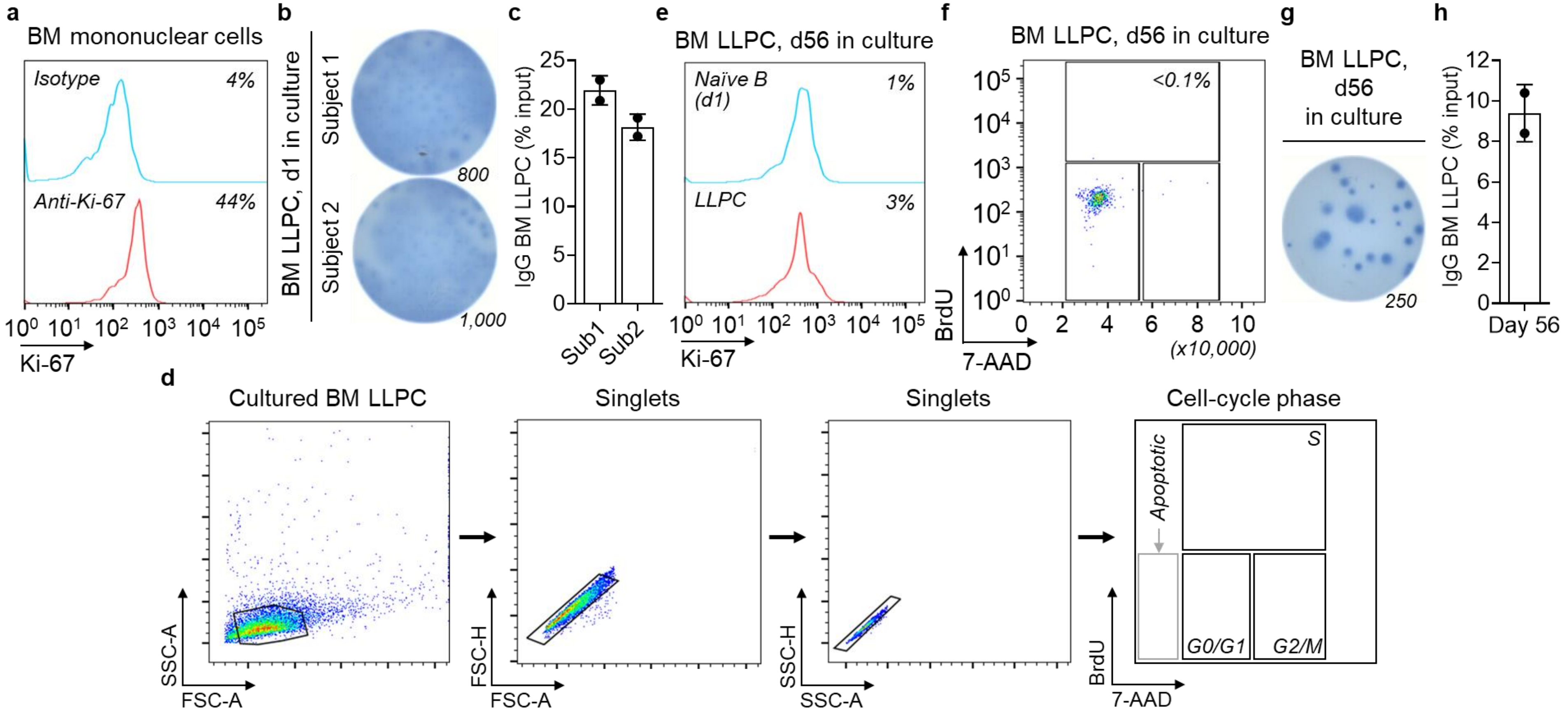
a
BM mononuclear cells
Isotype
4%
Anti-Ki-67
44%
Ki-67
b
BM LLPC, d1 in culture
Subject 1
Subject 2
800
1,000
c
IgG BM LLPC (% input)
Sub1
Sub2
e
BM LLPC, d56 in culture
Naïve B (d1)
1%
LLPC
3%
Ki-67
f
BM LLPC, d56 in culture
<0.1%
BrdU
7-AAD
(x10,000)
g
BM LLPC, d56 in culture
250
h
IgG BM LLPC (% input)
Day 56
d
Cultured BM LLPC
Singlets
Singlets
Cell-cycle phase
SSC-A
FSC-A
FSC-H
FSC-A
SSC-H
SSC-A
Apoptotic
S
G0/G1
G2/M
BrdU
7-AAD

Suppl. Fig. S5.

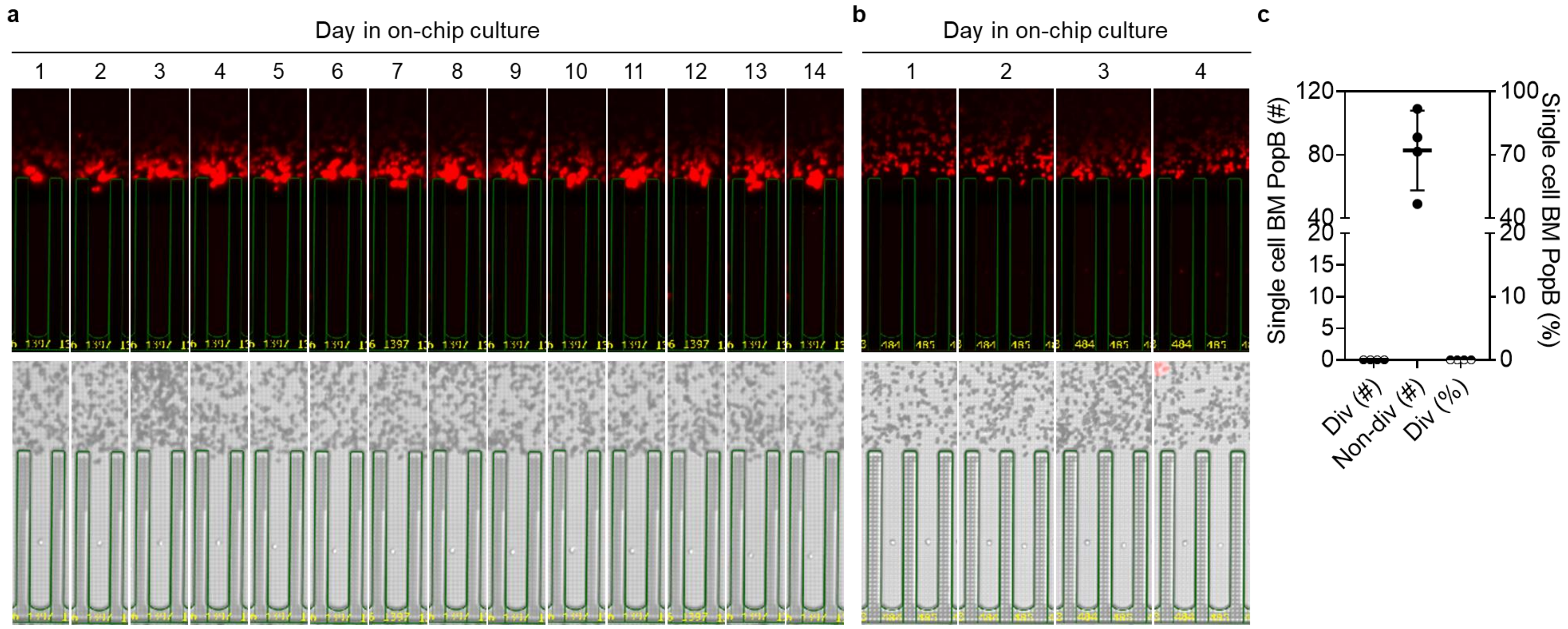
a
Day in on-chip culture
1
2
3
4
5
6
7
8
9
10
11
12
13
14
b
Day in on-chip culture
1
2
3
4
c
Single cell BM PopB (#)
Single cell BM PopB (%)
120
80
40
20
15
10
5
0
100
70
40
20
10
0
Div (#)
Non-div (#)
Div (%)

Suppl. Fig. S6.

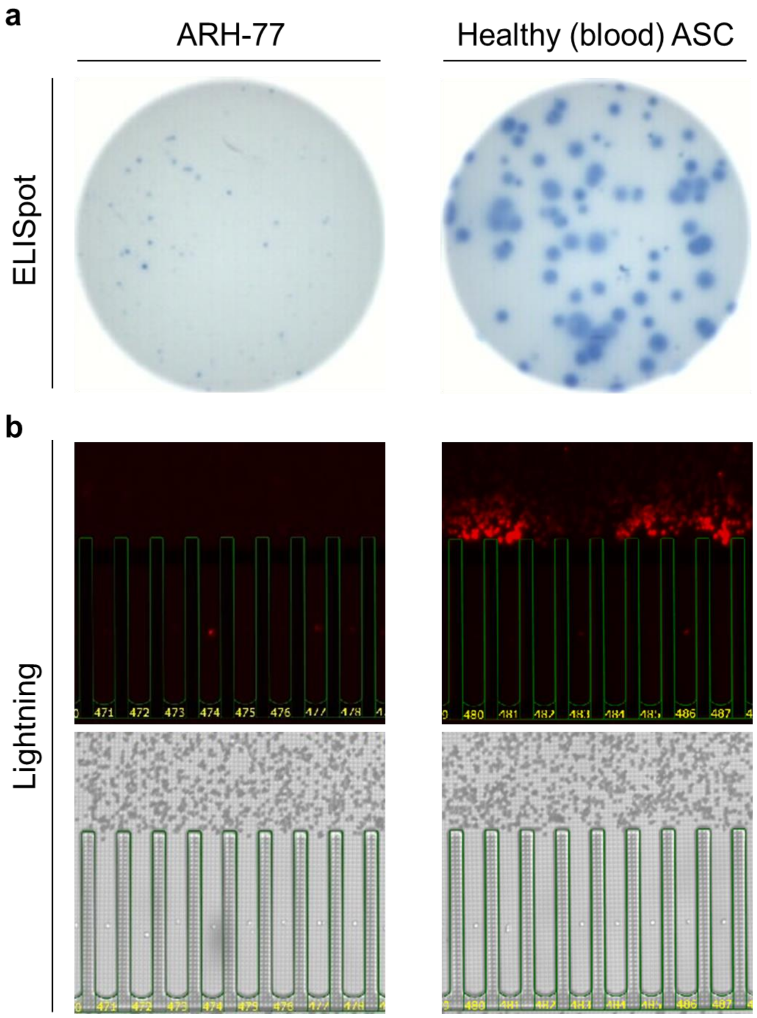
a
ARH-77
Healthy (blood) ASC
ELISpot
b
Lightning
471
472
473
474
475
476
480
486
487
